# Testing Mechanisms of Change for Text Message–Delivered Cognitive Behavioral Therapy: Randomized Clinical Trial for Young Adult Depression

**DOI:** 10.2196/45186

**Published:** 2023-07-11

**Authors:** Michael J Mason, J Douglas Coatsworth, Nikola Zaharakis, Michael Russell, Aaron Brown, Sydney McKinstry

**Affiliations:** 1 Center for Behavioral Health Research College of Social Work University of Tennessee Knoxville, TN United States; 2 Department of Biobehavioral Health Pennsylvania State University University Park, PA United States; 3 College of Social Work University of Kentucky Lexington, KY United States

**Keywords:** young adults, depression, SMS text message–delivered treatment, cognitive behavioral therapy, randomized clinical trial, mobile health treatment, mHealth treatment, mobile phone

## Abstract

**Background:**

Current psychiatric epidemiological evidence estimates that 17% of young adults (aged 18-25 years) experienced a major depressive episode in 2020, relative to 8.4% of all adults aged ≥26 years. Young adults with a major depressive episode in the past year are the least likely to receive treatment for depression compared with other age groups.

**Objective:**

We conducted a randomized clinical trial following our initial 4-week SMS text message–delivered cognitive behavioral therapy (CBT-txt) for depression in young adults. We sought to test mechanisms of change for CBT-txt.

**Methods:**

Based on participant feedback, outcome data, and the empirical literature, we increased the treatment dosage from 4-8 weeks and tested 3 mechanisms of change with 103 young adults in the United States. Participants were from 34 states, recruited from Facebook and Instagram and presenting with at least moderate depressive symptomatology. Web-based assessments occurred at baseline prior to randomization and at 1, 2, and 3 months after enrollment. The primary outcome, the severity of depressive symptoms, was assessed using the Beck Depression Inventory II. Behavioral activation, perseverative thinking, and cognitive distortions were measured as mechanisms of change. Participants were randomized to CBT-txt or a waitlist control condition. Those assigned to the CBT-txt intervention condition received 474 fully automated SMS text messages, delivered every other day over a 64-day period and averaging 14.8 (SD 2.4) SMS text messages per treatment day. Intervention texts are delivered via TextIt, a web-based automated SMS text messaging platform.

**Results:**

Across all 3 months of the study, participants in the CBT-txt group showed significantly larger decreases in depressive symptoms than those in the control group (*P*<.001 at each follow-up), producing a medium-to-large effect size (Cohen *d*=0.76). Over half (25/47, 53%) of the treatment group moved into the “high-end functioning” category, representing no or minimal clinically significant depressive symptoms, compared with 15% (8/53) of the control condition. Mediation analysis showed that CBT-txt appeared to lead to greater increases in behavioral activation and greater decreases in cognitive distortions and perseverative thinking across the 3-month follow-up period, which were then associated with larger baseline to 3-month decreases in depression. The size of the indirect effects was substantial: 57%, 41%, and 50% of the CBT-txt effect on changes in depression were mediated by changes in behavioral activation, cognitive distortions, and perseverative thinking, respectively. Models including all 3 mediators simultaneously showed that 63% of the CBT-txt effect was mediated by the combined indirect effects.

**Conclusions:**

Results provide evidence for the efficacy of CBT-txt to reduce young adult depressive symptoms through hypothesized mechanisms. To the best of our knowledge, CBT-txt is unique in its SMS text message–delivered modality, the strong clinical evidence supporting efficacy and mechanisms of change.

**Trial Registration:**

ClinicalTrials.gov NCT05551702; https://clinicaltrials.gov/study/NCT05551702

## Introduction

### Background

In 2020, young adults (ages 18-25 years) in the United States experienced more major depressive episodes than other age groups and were the least likely to receive treatment for depression (eg, talking with a health provider or taking prescription medication) [1].

Even when the rates for the treatment of depression rose nationwide, the rates for the treatment of depression for young adults rose at a significantly lower rate [2]. Paradoxically, more young adults with depression report greater perceived unmet need for mental health services than other age groups [1]. From 2011 to 2019, the most common reason that young adults report for not receiving treatment is cost, followed by “not knowing where to go to receive treatment” [3]. This unmet treatment need has been consistent over the last decade and places this group at an increased risk for substance use disorders, possibly indicating self-medication with substances to reduce depressive symptoms [4].

### Mobile Health Treatments

Creative mental health treatment strategies are needed to reach young adults to address this unmet need. Digitally delivered mobile health (mHealth) treatments (or mHealth interventions) show promise for reaching young adults and serving as a clinical tool to address depression in young adults. Because mHealth interventions can increase large-scale access to evidence-based treatments, some countries such as New Zealand are integrating these approaches into their national health service infrastructure [5]. The rapid development and use of these treatments is a promising step for addressing psychiatric disorders. However, there is reason to be cautious regarding the science behind these efforts. In particular, rigorous studies on 2 important features of mHealth interventions are lacking in the research literature: testing treatment dosage and clinical mechanisms of change. First, understanding the appropriate dosage for mHealth interventions is critical to advance an empirical database that has maximum clinical utility. Knowing the correct dosage (how much treatment is needed to be delivered to meet the treatment goals of particular patients) is a primary goal of clinical research. In a recent meta-analysis that assessed the relationship between mHealth intervention length and intervention effect, Lu et al [6] found that interventions of at least 7 weeks’ duration had larger effect sizes on anxiety symptom reduction. However, for depression treatment, the optimal dosage remains unclear. Second, understanding the mechanisms of behavior change has not been adequately studied in mHealth interventions. Digital interventions have tremendous potential for identifying mechanisms of behavior change because these can be cost-effectively delivered to large enough samples to sufficiently power studies to detect mechanisms of change, the content is delivered reliably and precisely, and the manipulation of the hypothesized mediators can be strategically sequenced within a study design [7]. Unfortunately, many mHealth studies lack scientific rigor (eg, the lack of a priori hypothesized causal mechanisms of change coupled with an appropriate design), which is necessary to conduct mediational analyses [7]. In addition, although the delivery of mHealth interventions for mental disorders continues to expand, accompanying rigorous randomized clinical trials, including those that test dosage and mechanisms, have not kept pace with this growth [8].

### Clinical Foundation

This study was conducted to better understand the mechanisms of SMS text message–delivered cognitive behavioral therapy (CBT-txt) as well as the acceptability of the treatment length. Our recent randomized clinical trial treating 102 young adults in the United States with at least moderate depressive symptomatology found that CBT-txt was acceptable, feasible, and efficacious compared with a waitlist control condition [9]. This 2-month trial had 4 weeks of treatment and tested a single mediator, behavioral activation, with assessments at baseline and at 1- and 2-month follow-ups. The treatment length mirrored the duration of our successful text-delivered cannabis use disorder treatment [10]. The initial CBT-txt trial found that the strongest treatment effect appeared at the 1-month follow-up (immediately after the treatment ended), particularly for participants who began with severe depressive symptoms. Mediation analysis revealed significant indirect treatment effects of increases in behavioral activation on reducing depressive symptoms, suggesting a mechanism of change [9,11]. Based upon these promising findings, we examined the treatment satisfaction data from this study, which revealed participants’ interest in receiving more treatment content and an increase in the duration of the intervention. Accordingly, we expanded CBT-txt from 4 to 8 weeks, which is consistent with the average mHealth dosage of 7 to 8 weeks [6,12]. We also expanded the treatment’s clinical content to include cognitive distortions and perseverative thinking, 2 additional candidate mediators common to cognitive behavioral therapy (CBT) treatment of depression [13-15].

### This Study

Four hypotheses were tested for this follow-up study. The first hypothesis was that participants allocated to the treatment condition would show greater reductions in their depressive symptoms immediately following treatment (2 months after enrollment) and at the 3-month follow-up than participants in the waitlist control condition. Because treatment response has been linked to baseline symptom severity [9,16], we also tested whether treatment effects were moderated by the participants’ baseline severity level. The second hypothesis was that treatment effects would be mediated by behavioral activation, such that increases in behavioral activation would reduce depressive symptoms in the treatment condition relative to the controls. The third hypothesis was that treatment effects would be mediated by cognitive distortions, such that decreases in cognitive distortions would reduce depressive symptoms in the treatment condition relative to controls. The fourth hypothesis was that treatment effects would be mediated by perseverative thinking, such that decreases in perseverative thinking would reduce depressive symptoms in the treatment condition relative to the controls. In addition to testing these 4 hypotheses, we were interested in comparing the results (effect sizes and treatment response over time) from this 8-week intervention with those from our prior trial of the 4-week intervention.

## Methods

### Procedures

In total, 103 young adults (ages 18-25 years) were recruited and enrolled in a 3-month randomized clinical trial. Participants were recruited using age-targeted advertising on Facebook and Instagram for young adults residing in the United States. Recruitment occurred over a 7-week period from July 6 to August 28, 2022. The study was advertised to all those within the study age range (18-25 years) across the United States who used Facebook and Instagram. With the aim of recruiting a geographic, racial and ethnic, and socioeconomically diverse sample, we placed no restrictions on advertisements other than age, used advertisements with a variety of images featuring people of different racial groups, and used a variety of placements (eg, feeds, stories, and reels) to reach the widest swath of potential participants. Interested individuals were directed to a study website where they would read more about the study and answer eligibility screening questions on their phones.

The study was registered at ClinicalTrials.gov (identifier NCT05551702).

### Design

The study design was a 2-arm randomized clinical trial with participants allocated to either the experimental condition or the waitlist control condition. Eligibility requirements were (1) age between 18 and 25 years; (2) a score of at least 10 on the Patient Health Questionnaire–9 (PHQ-9) [17], indicating at least moderate depressive symptom severity; (3) access to a smartphone; (4) fluent in English; (5) have not received treatment for depression in the past 3 months; and (6) did not endorse suicidal ideation (SI) on the PHQ-9 measure. All interested participants completed a screening questionnaire assessing eligibility. Those who screened positive for suicide and those who did not meet the study criteria were immediately referred to local and national mental health resources. To ensure that participants who did not qualify for the study still had access to care, participants who were not eligible based on the endorsement of SI had the option of speaking with a project staff member who is a licensed mental health professional for assistance with referral to care. Individuals who wanted to talk with a licensed mental health staff member were contacted within 1 business day and provided contact information to at least 3 mental health providers in their area, including at least 1 provider who offered sliding scale or safety net (ie, no cost) services. In addition, individuals were given the contact information to the National Alliance on Mental Illness location in their area. The National Alliance on Mental Illness provides additional mental health resources, including no-cost support groups for individuals living with mental illness [18].

### Remote Data Collection Quality Control

Data were reviewed carefully on a daily basis for quality control. Beginning July 26, 2022, we noticed increased screening activity with similar name patterns. After carefully reviewing the screening data, including the name, mailing address, IP address, phone number with area code and service carrier, and survey responses, we determined that 17 enrolled cases may have been fraudulent. We reached out via text, phone, and email to these 17 cases to confirm their identity but none of the participants responded. All 17 cases were administratively removed from the study on July 28, 2022. We enacted other procedures, such as using Qualtrics (Qualtrics), the web-based survey program that flags responses likely to be from bots, attempts to prevent multiple submissions from a single respondent, and assigns a fraud score indicating the likelihood of a response being fraudulent [19]. Furthermore, at screening, participants must enter a unique phone number, which is then verified using a Twilio mobile phone app.

Eligible individuals were instructed to complete the baseline survey on their phones, where upon completion, they were randomized to either the treatment or waitlist control condition. Randomization was automated by Qualtrics as part of the baseline survey. Randomization was stratified by sex using block randomization with a fixed block size of 10 to reduce bias during randomization and to ensure equal representation of sex across both conditions. The participants completed follow-up assessments at the 1-, 2-, and 3-month follow-ups. Waitlist condition participants were eligible to receive the treatment texts upon the completion of the 3-month follow-up survey. Participants who completed the screening, baseline, and all follow-up assessments received US $150 in Amazon eGift cards.

### Text-Delivered Treatment: CBT-txt

#### Overview

CBT-txt was adapted from an evidenced-based, in-person CBT treatment manual [20] shown to be effective in reducing depressive symptoms [21-23] and adaptable to digital formats [24]. CBT-txt focuses on empowering participants to understand how thoughts, activities, and other people affect their moods. CBT-txt is a fully automated SMS text message–delivered program that initiates a conversation at predetermined times and requires a participant response to activate the subsequent text message. Tailored messages are sent based on the participant’s responses or ratings of their depression. For example, a participant may be asked about their engagement with CBT skills and are given 3 response choices: a, b, or c. Each response option activates a different CBT-txt message providing tailored responses. The treatment provides individualized text-based conversations every other day over the course of the intervention.

#### Theoretical Underpinnings of CBT-txt

The theory underlying CBT-txt is the Generic Cognitive Model, which specifies common cognitive and behavioral processes associated with disorders such as depression [25]. These processes manifest along a continuum from normal adaptive functioning to psychopathology. As individuals experience environmental, psychological, and social stimuli, their attentional response system is activated to determine an adaptive response. The attentional response activates schemas, defined as internally stored representations of stimuli that form underlying structures for organizing perceptions of the world [26]. When the schema is maladaptive or disproportionate relative to the stimuli, the individual experiences psychological problems that can escalate into a psychiatric disorder.

#### Clinical Structure of CBT-txt

We expanded our initial 4-week CBT-txt treatment [9] into an 8-week intervention. CBT-txt intervention content is guided by CBT core mechanisms [13] and is sequenced using the in-person treatment manual of Muñoz et al [20]. CBT-txt is organized around eight topical areas delivered across 8 weeks as indicated in Table 1: (1) introduction to CBT, (2) automatic thoughts, (3) behavioral activation, (4) automatic thoughts and health, (5) perseverative thinking, (6) cognitive distortions, (7) more on behavioral activation, and (8) social support and summary.

**Table 1:** SMS text message–delivered cognitive behavioral therapy (CBT) content and structure (474 texts over 8 weeks; mean 14.8 texts per day, every other day).

Week	Days, n	Texts, n	Treatment content
1	4	62	How we think about depression (Intro to CBT, moods, thoughts, feelings)
2	4	72	How thoughts affect moods (Automatic thoughts; ABCD method)
3	4	62	How activities affect moods (Behavioral activation)
4	4	57	How thoughts affect our habits and health (ABCD method & health)
5	4	55	How repetitive negative thinking affects moods (Perseverative thinking)
6	4	53	How cognitive distortions affect our moods (Cognitive distortion)
7	4	59	How activities, goals, and values affect our moods (Behavioral activation)
8	4	54	How other people affect moods (Social support and moods); Summary

Participants assigned to the CBT-txt intervention condition received 474 texts delivered every other day over a 64-day period, averaging 14.8 texts per treatment day. Participants indicated the time of day they would like to receive the intervention texts. Intervention texts were delivered via a web-based automated SMS text messaging platform called TextIt. Project staff programmed TextIt to deliver intervention texts and extract data from the web-based survey platform Qualtrics to automatically personalize intervention texts. Texts were individualized based on data provided by participants in the baseline survey as well as throughout the treatment period.

In addition to the scheduled intervention messages, participants could access automated booster messaging to provide on-demand supportive messages by texting “4MOOD” at any time, as needed. The “4MOOD” messages are organized around topics (eg, cognitive techniques and positive activities) such that participants select a category of message to receive each time they text “4MOOD” for additional support. All text messages end with the response, “If this is a crisis call 911.” If participants want to find out about receiving professional help, they choose option 4 and the program texts them a link to a list of national mental health services, suicide prevention hotlines, and a child and adult abuse hotline. This list includes a link to access Substance Abuse and Mental Health Services Administration’s treatment locator, which offers a database of treatment providers that can be filtered by location. Textbox 1 provides example SMS text messages of CBT-txt as well as an example booster message.

**Example of a texting conversation over 1 day. (The italicized text is autopopulated by programming and is programming logic; it is not shown to the participant. This limited example of SMS text message–delivered cognitive behavioral therapy does not provide the context of previous texting conversations or subsequent conversations that are built on past conversations.)**

1. Hi *name*. Before we start, how is your mood right now? (0=very good-10=very depressed) Text 0-10
2. Let’s look at how thoughts, what we tell ourselves, affect our mood. We are usually not aware of these negative thinking patterns that influence our mood
3. Text *selftalk* any time to read the 10 common negative thinking patterns. We believe these thoughts because we think them and don’t challenge them. Try it now!
4. We actually fool ourselves and create more bad feelings about things that are *simply not true.* Our depressed thinking keeps us in this cycle.
5. Study the list carefully, see which patterns you use. We will refer to these often as these serve as a guide to identify and change our thinking and mood. Text OK
6. Looking at the list, identify at least 1 of the thinking patterns that you’ve used recently. Text back the number of the pattern. Text 1-10 or UNSURE

*If 1-10:*

Good job identifying a pattern. *all or nothing thinking* is very common as are most of these. We’re unaware of these patterns so we call them automatic thoughts

So, what was the situation that led you to use *all or nothing thinking*? Please describe. Text__

*IF UNSURE:*

Ok, Look at the list again & think about this some more: http://selftalk.mjmood.com Text READY when you’re ready to discuss. Text UNSURE if you’re still unsure.

*If READY, repeat #6*

*IF UNSURE:*

Ok, we know it can be hard to look at the way we talk to ourselves honestly. Remember that the more you put into this program the more you will get out of it.

1. Carefully observe your automatic thoughts the next few days. Try to challenge them as not accurate or facts, but a result of your being in a depressed mood
2. This is a critical skill for you to learn. It seems simple, but when you practice this you’ll be surprised at how much this can change how you feel.
3. Thanks *name*! That ends today’s texts. Need mood support? Text: *4MOOD*. This is not being read immediately, if this is a crisis call 911

### Fidelity of CBT-txt

We followed previous successful research in developing text-delivered interventions derived from in-person treatments [9,27-29]. The following four steps were used to ensure that the core mechanisms of CBT were incorporated into CBT-txt: (1) selected an evidence-based treatment manual as an adaptation source [20] as well as primary CBT source materials [26], (2) developed texts to match the treatment manual content and structure [20], (3) cross-checked the texts against the core mechanisms of CBT for depression [13], and (4) applied a CBT fidelity scale [30] to rate the texts with an outside expert, Dr John Curry, who provided independent quality assurance scoring. Dr John Curry is a professor of Psychiatry and Behavioral Sciences at Duke University and is a nationally recognized expert in CBT who wrote the protocol for the Treatment of Adolescents with Depression Study [31]. Finally, we applied the results of the fidelity review (average rating of 4 out of 5, with 5 being excellent) and made revisions as needed (provided more overview and summary content).

### Participant Safety Protocol

We instituted a participant safety protocol that reviewed all incoming texts for crisis-related words both automatically (ie, autotext review) and with staff reading every text from all participants at least once per day. If participants indicated SI on the Beck Depression Inventory-II (BDI-II) or if their follow-up BDI-II (see the *Measures* section) score increased, they were automatically sent a supportive SMS text message that included national resources. Furthermore, a licensed mental health professional on the staff texted, emailed, and called participants falling into these at-risk categories to ensure safety, ensure the availability of resources, and provide local treatment resources and general support.

### Measures

#### Demographics

Participant age, sex, race and ethnicity, socioeconomic status, family history of depression, and current use of antidepressant medication were collected at the baseline assessment.

#### Acceptability and Engagement

Participants’ acceptability of the intervention was measured via participant-reported satisfaction with the intervention as well as passively collected engagement with the intervention content and features. Satisfaction was assessed using a measure developed in our past work [32]. Two items assessed whether the number of days on which texts were received and the number of texts received each day were (1) *too few*, (2) *just right*, or (3) *too many*. An additional 2 items measured whether the texts were easy to understand and complete and whether the “4MOOD” booster messages were a helpful option. In addition, 5 items measured perceptions of the helpfulness of the intervention content (Cronbach α=.88), and 6 items assessed participants’ practice of the intervention skills taught (Cronbach α=.78). All of these items were rated on a 5-point Likert scale (1=*strongly disagree* to 5=*strongly* *agree*). Engagement with the intervention was measured using data passively collected that were programmed to be automatically gathered during the administration of the intervention, including the number of intervention texts a participant responded to each day and the number of booster SMS text messages requested. Intervention completion was defined as responding to ≥95% of all intervention texts (at least 192 responses out of a total of 198 responses across all intervention days).

#### Screen of Depression Symptoms

The PHQ-9 was used to determine eligibility [17]. The PHQ-9 is a questionnaire consisting of all 9 criteria for major depressive disorder (MDD). Responses range from “not at all” (score=0) to “nearly every day” (score=3). Item scores are summed for a total score ranging from 0 to 27. The PHQ-9 has good validity, test-retest reliability, and internal consistency. Cronbach α in this study’s sample was .50.

#### Depression Symptoms and Severity

Baseline and follow-up assessment of depression severity was assessed with BDI-II [33]. There are 21 items, each corresponding with a symptom of depression, and possible scores on each item range from 0 to 3. The scores are summed to obtain a single severity score. A score of 0 to 13 indicates none or minimal depressive symptoms, a score of 14 to 19 indicates mild depressive symptoms, a score of 20 to 28 indicates moderate depressive symptoms, and a score of ≥29 indicate severe depressive symptoms. Cronbach α in this study’s sample at each assessment were .83, .89, .92, and .92, respectively.

#### Behavioral Activation

Behavioral activation was measured using the Behavioral Activation for Depression Scale, Short Form [34]. The Behavioral Activation for Depression Scale, Short Form is a 9-item scale used to assess activation toward goals during the treatment for depression. Items are scored from 0 to 6, and higher scores indicate greater activation toward goals and less avoidance of tasks. Cronbach α in this study’s sample at each assessment were .70, .70, .82, and .81, respectively.

#### Perseverative Thinking

Repetitive negative thinking was measured using the Perseverative Thinking Questionnaire (PTQ) [35]. The PTQ is a 15-item questionnaire used to characterize respondents thinking about negative experiences or problems. Items are scored on a 5-point scale from 0=*never* to 4=*almost always* and are summed with higher scores indicating more repetitive negative thinking. Cronbach α in this study’s sample at each assessment was .94, .95, .93, and .97, respectively.

#### Cognitive Distortion

Cognitive distortion was measured using the Cognitive Distortions Scale (CDS) [36]. The CDS assesses 10 types of thinking biases (eg, catastrophizing or all-or-nothing thinking). Participants rate the frequency of their use of each type of thinking. Items are scored on a 7-point scale from 0=*never* to 7=*all the time* and are summed with higher scores indicating more cognitive distortion. Cronbach α in this study’s sample at each assessment was .92, .94, .93, and .95, respectively.

### Statistical Analyses

#### Latent Change Score Modeling

Latent change score (LCS) analyses were conducted in a structural equation modeling framework using the *lavaan* package in R (R Foundation for Statistical Computing). LCS was used instead of more commonly used modeling methods, such as latent growth modeling and repeated measures ANOVA, because it offers 2 unique capabilities. First, it allows for the estimation of wave-to-wave change in mediators and outcomes adjusted for previous levels, which controls for regression to the mean. Second, it does not force the pattern of change to follow a prespecified shape; wave-to-wave changes are allowed to freely vary across time [37,38]. LCSs are created by (1) specifying an autoregression of each score on its immediately previous time point while fixing the coefficient to 1 and (2) specifying a latent factor with a loading of 1 on the current time point to capture the difference. These LCSs are then used as dependent variables in regressions testing for CBT-txt intervention effects. BDI-II depression scores were centered on their pretreatment mean; CBT-txt was also centered (CBT-txt=0.51; control=−0.49). This centering strategy facilitates the interpretation of model intercepts as a change from the previous month for a person with average pretreatment levels of depression, averaged across conditions. All LCS regressions controlled for both pretreatment levels of depression and prior-month levels of depression to account for the likely association between pretreatment severity and posttreatment change.

#### Mediation Analysis

Mediation was also tested in LCS models. LCSs were created for (1) the baseline to 3-month change in the mediator (Behavioral Activation for Depression Scale, CDS, and PTQ in separate models) and (2) the baseline to 3-month change in the outcome (BDI-II). Pretreatment mediator and BDI-II scores at baseline were included as covariates in the model. The change in mediators was regressed on CBT-txt (and baseline covariates) to create the *a* path; the change in BDI-II was regressed on the change in the mediator (the *b* path) and CBT-txt (the direct effect or *c’* path). The significance of the indirect effect (a*b) was tested using bias-corrected bootstrapped CIs with 10,000 bootstrap draws. The indirect effect tests the hypothesis that CBT-txt influences BDI-II through the mediator.

### Ethics Approval

All procedures were approved by the institutional review board of The University of Tennessee (approval UTK IRB-20-06164-FB).

## Results

### Demographic Results

**Figure 1:**
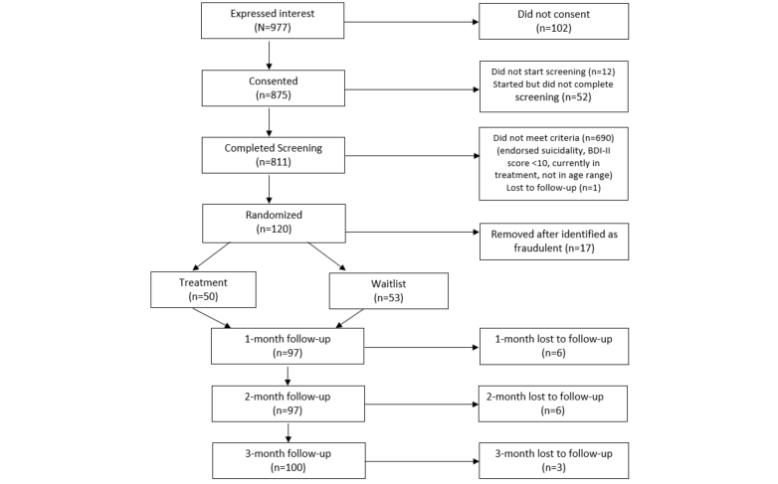
CONSORT diagram for study participant flow and condition allocation.

Our sample size was 103. The participants’ mean age was 22 (SD 2.2) years and 84.5% (87/103) were female, and they resided in 34 different states in the United States and the District of Columbia. The sample comprised 15.5% (16/103) Asian, 3.9% (4/103) Black or African American, 7.8% (8/103) Hispanic or Latino, 8.7% (9/103) more than 1 race, and 63.1% (65/103) White participants. While not used analytically, college student status, childhood socioeconomic status (“Has your family ever received food assistance, such as free or reduced lunch, or SNAP benefits?”), and past and current use of antidepression medication were described to characterize the sample. Slightly more than half (55/103, 54.5%) were not enrolled in college part time or full time. Approximately one-third (35/103, 34%) of the participants endorsed a family history of government food assistance, and 42.7% (44/103) of participants had been prescribed antidepressant medication at some time in their life but only 20.4% (21/103) were currently prescribed antidepressant medication. Figure 1 provides details of the enrollment and allocation process via the CONSORT (Consolidated Standards of Reporting Trials) diagram.

### Acceptability and Engagement

Participants endorsed levels of acceptability and engagement similar to our previous pilot of CBT-txt for young adult depression. Two-thirds (69/103, 67%) of the participants completed the intervention (responding to ≥95% of intervention texts), although somewhat lower than in our previous pilot (85%) [9] but still substantially higher than the average response rates for unguided internet-delivered CBT (40%) [39]. On average, participants completed 163.6 (SD 60.2) texts of the total potential 198 responses. Slightly more participants (37/50, 74%) completed the first month of texts (111 total potential responses; mean 95.3, SD 30.6) than the second month of texts (33/50, 66%; mean 68.3, SD 30.9). The participants endorsed high levels of satisfaction with the treatment content and features. More participants agreed or strongly agreed (35/44, 80%) that the intervention texts were more helpful than in our first pilot study (77%), that the number of days of texts received per day was “just right” (32/42, 79%) than previously (76%), and that the number of texts per day was “just right” (36/42, 86%) than in the previous study (72%) [9]. The overall mean of the helpfulness subscale (mean 4.0, SD 0.7) was very similar to that of the prior trial (mean 3.9, SD 0.8) [9]. However, fewer participants (36/44, 82%) reported that the texts were easy to understand and complete than those in the previous trial (93%) [9]. The participants endorsed moderate levels of implementing the skills learned (mean 3.2, SD 0.7), very similarly to our previous study [9]. Slightly more (32/44, 73%) number of participants agreed that the booster messages were a useful option compared with our prior study (68%) [9]. However, fewer participants (20/50, 40%) used booster messages than the previous trial (42%) [9].

### LCS Results

#### Main Effect of CBT-txt on Depression

**Figure 2:**
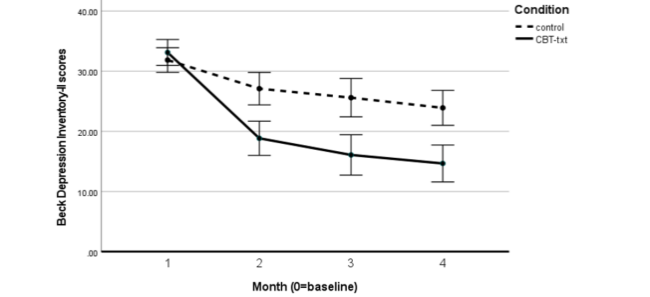
Mean scores of the Beck Depression Inventory-II scores over time by condition. CBT-txt: SMS text message–delivered cognitive behavioral therapy.

Figure 2 shows the Beck Depression Inventory means over time for the CBT-txt and control groups. Significant treatment–control group differences were observed at each of the 3 postenrollment follow-ups (*P*<.001 at each follow-up). No treatment–control group difference was seen in pretreatment depression, supporting balance across groups in baseline levels. Table 2 shows the results of the LCS model testing whether the monthly changes differed between the CBT-txt and control groups. At each of the 3 follow-ups, young adults in the CBT-txt group showed significantly larger decreases in depression than those in the control group, producing a medium to large effect size (Cohen *d*=0.76).

**Table 2:** Latent change score model results testing SMS text message–delivered cognitive behavioral therapy (CBT-txt) efficacy for depression treatment.

			Estimate	SE	*Z* value	*P* value	95% CI
**Baseline depression level**							
	Intercept		0.00	0.89	0.00	>.99	−1.75 to 1.75
	CBT-txt		−0.22	1.78	−0.12	.90	−3.71 to 3.27
**Change**							
	**Baseline–1 month**						
		Intercept	−*9.17*^a^	0.86	−10.63	<.001	−10.86 to −7.48
		CBT-txt	−*9.02*	1.73	−5.22	<.001	−12.40 to −5.63
		Baseline depression level	−*0.51*	0.10	−5.33	<.001	−0.70 to −0.32
	**1 month–2 month**						
		Intercept	−*4.65*	1.20	−3.88	<.001	−7.00 to −2.30
		CBT-txt	−*3.79*	1.82	−2.08	.04	−7.36 to −0.21
		1-month depression Level	−*0.27*	0.10	−2.87	.004	−0.46 to −0.09
		Baseline depression level	*0.20*	0.10	1.97	.049	0.00 to 0.40
	**2 month–3 month**						
		Intercept	−*6.68*	1.27	−5.25	<.001	−9.18 to −4.19
		CBT-txt	−*4.64*	1.90	−2.44	.02	−8.37 to −0.91
		2-month depression level	−*0.50*	0.09	−5.93	<.001	−0.67 to −0.34
		Baseline depression level	0.07	0.11	0.69	.49	−0.13 to 0.28

^a^Italicized text indicates *P*<.05.

#### Clinical Significance

We used 2 measures of clinical significance. First, the number of participants at the end of the trial with high-end state functioning as defined by Jacobson and Truax [40] was measured. High-end state functioning is defined as a participant having a BDI-II score between 0 and 13. By examining the BDI-II severity levels based on total scores (*none to minimal depressive symptoms*=0-13, *mild depressive symptoms*=14-19, *moderate depressive symptoms*=20-28, and *severe depressive* symptoms=29-63), treatment responses can be understood clinically. Thus, scores between 0 and 13 represent high-end state functioning owing to the elimination of symptoms used to classify participants with MDD. Over half (25/47, 53%) of the treatment group moved to the “none to minimal” category, compared with 15% (8/53) of the control group. Second, we used a reliable change index (RCI), which is a measure of how much change has occurred during the course of treatment [40]. Our study produced an RCI of 4.46 with 95% confidence, which is twice the minimum required RCI of 1.96. This RCI represents 2 SDs of clinical change for the treatment group based on the baseline to 3-month follow-up BDI-II scores. Table 3 displays the depression severity level percentages at the 3-month assessment for comparison by experimental conditions.

**Table 3:** Depression severity level (BDI-II categories) percentages at 3 months by condition.

Depression severity levels	CBT-txt^a^	Control
None to minimal	53%	15%
Mild	17%	5%
Moderate	13%	45%
Severe	17%	34%

#### Main Effect of CBT-txt on Mechanisms

**Figure 3:**
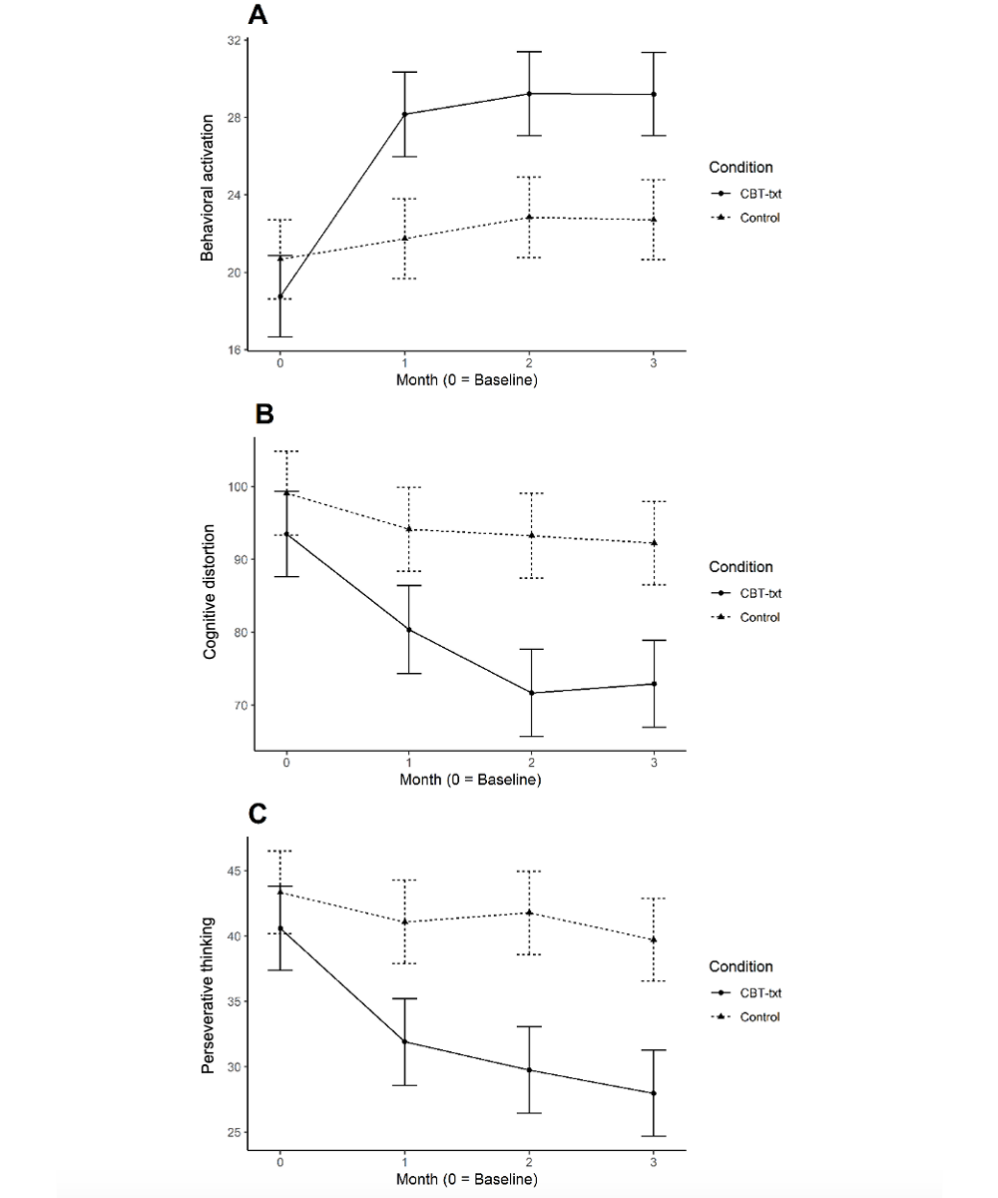
Mean scores of (A) behavioral activation (Behavioral Activation for Depression Scale), (B) cognitive distortion (Cognitive Distortions Scale), and (C) perseverative thinking (Perseverative Thinking Questionnaire), measures overtime by condition. BDI: Beck Depression Inventory; CBT-txt: SMS text message–delivered cognitive behavioral therapy.

Figure 3 shows the means of the hypothesized treatment mechanisms (behavioral activation, cognitive distortion, and perseverative thinking in panels A, B, and C, respectively) over time for the CBT-txt and control groups. None of the pretreatment means were significantly different between the CBT-txt and control groups. Significant treatment–control group differences were observed at each of the follow-up waves for each of the 3 mechanisms, with higher behavioral activation and lower cognitive distortions and perseverative thinking observed among CBT-txt versus control participants. Tables S1-S3 in Multimedia Appendix 1 show the LCS results for the 3 mechanisms. The largest and most consistently significant treatment effects were observed at the 1-month follow-up. Significantly larger 1- to 2-month decreases in cognitive distortions and perseverative thinking were seen for CBT-txt versus control participants; no significant treatment–control group differences in 1- to 2-month change in behavioral activation were seen. No significant treatment–control differences in the 2- to 3-month change were observed for any of the mechanisms.

#### LCS Mediation Results

Table 4 shows *a*, *b*, *c’*, indirect, and total effects for mediation models testing indirect effects of CBT-txt on change in depression through change in the 3 hypothesized mechanisms (Tables S4-S6 in Multimedia Appendix 1 show full model results for LCS mediations). Significant direct and indirect effects of CBT-txt on depression were observed in all 3 models. CBT-txt appeared to lead to greater increases in behavioral activation and greater decreases in cognitive distortions and perseverative thinking across the 3-month follow-up period, which were then associated with larger baseline to 3-month decreases in depression. The size of the indirect effects was substantial: 57%, 41%, and 50% of the CBT-txt effect on changes in depression were mediated by changes in behavioral activation, cognitive distortions, and perseverative thinking, respectively.

Models testing all mediators simultaneously were also conducted and both combined and independent indirect effects were estimated (Table 5). The combined indirect effect, representing the combined action of all 3 mediators, was significant (estimate=−6.07, 95% CI −8.95 to −3.26) and explained 63% of the treatment effect on depression. Independent indirect effects were nonsignificant for behavioral activation (estimate=−2.09, 95% CI −4.92 to 0.24) and cognitive distortions (estimate=−1.14, 95% CI −3.40 to 1.20) but were significant for perseverative thinking (estimate=−2.83, 95% CI −5.80 to −0.57). Behavioral activation, cognitive distortions, and perseverative thinking independently explained 22%, 12%, and 30% of the CBT-txt effect on depression, respectively.

**Table 4:** Latent change score mediation model results testing SMS text message–delivered cognitive behavioral therapy (CBT-txt) efficacy for depression through mediators.

				Estimate		SE		*Z* value		*P* value		95% CI
**CBT-txt > behavioral activation > depression**												
	**Paths**											
		CBT-txt > BADS^a^ change (*path* *a*)	*7.03* ^b^		1.68		4.20		<.001		3.75 to 10.30	
		BADS change > depression change (*path b*)	−*0.75*		0.11		−6.78		<.001		−0.96 to −0.53	
		CBT-txt > depression change (*path c′*)	−*4.01*		1.83		−2.19		.03		−7.69 to −0.49	
Indirect effect (a*b)				−*5.27*		—^c^		—		—		−8.69 to −2.54
Total effect (a*b + *c’*)				−*9.28*		—		—		—		−13.19 to −5.09
Percent mediated ([indirect/total] × 100%)				56.8		—		—		—		
**CBT-txt > cognitive distortion > depression**												
	**Paths**											
		CBT-txt > CDS^d^ change (*path* *a*)	−*15.98*		4.06		−3.94		<.001		−23.99 to −7.92	
		CDS change > depression change (*path b*)	*0.25*		0.04		6.30		<.001		0.17 to 0.32	
		CBT-txt > depression change (*path c′*)	−*5.67*		2.11		−2.69		.007		−9.85 to −1.58	
Indirect effect (a*b)				−*4.01*		—		—		—		−6.51 to −2.04
Total effect (a*b + *c’*)				−*9.68*		—		—		—		−13.67 to −5.35
Percent mediated ([indirect/total] × 100%)				41.4		—		—		—		
**CBT-txt > perseverative thinking > depression**												
	**Paths**											
		CBT-txt > PTQ^e^ change (*path* *a*)	−*10.33*		2.40		−4.30		<.001		−14.95 to −5.63	
		PTQ change > depression change (*path b*)	*0.50*		0.06		8.43		<.001		0.38 to 0.61	
		CBT-txt > depression change (*path c′*)	−*5.18*		1.79		−2.89		.004		−8.64 to −1.57	
Indirect effect (a*b)				−*5.12*		—		—		—		−8.05 to −2.66
Total effect (a*b + *c’*)				−*10.30*		—		—		—		−14.02 to −6.19
Percent mediated ([indirect/total] × 100%)				49.7		—		—		—		—

^a^BADS: Behavioral Activation for Depression Scale.

^b^Italicized text indicate *P*<.05.

^c^Not available.

^d^CDS: Cognitive Distortions Scale.

^e^PTQ: Perseverative Thinking Questionnaire.

**Table 5:** Latent change score mediation model results testing SMS text message–delivered cognitive behavioral therapy (CBT-txt) efficacy for depression through multiple mediators.

Paths	Estimate	SE	*Z* value	**P* value*	95% CI
CBT-txt > BADS^a^ 2-month change (*path* *a1*)	*7.29* ^b^	1.68	4.34	<.001	3.99 to 10.63
CBT-txt > CDS^c^ 2-month change (*path* *a2*)	−*18.72*	3.75	−4.99	<.001	−26.40 to −11.63
CBT-txt > PTQ^d^ 2-month change (*path* *a3*)	−*11.28*	2.33	−4.85	<.001	−16.05 to −6.83
BADS 2-month change > depression 3-month change (*path b1*)	−0.29	0.16	−1.80	.07	−0.57 to 0.06
CDS 2-month change > depression 3-month change (*path b2*)	0.06	0.06	1.02	.31	−0.07 to 0.17
PTQ 2-month change > depression 3-month change (*path b3*)	*0.25*	0.11	2.34	.02	0.04 to 0.46
CBT-txt > depression 3-month change (*path c′*)	−3.52	2.12	−1.66	.10	−7.66 to 0.66
Indirect effect, BADS (a1*b1)	−2.09	—^e^	—	—	−4.92 to 0.24
Indirect effect, CDS (a2*b2)	−1.14	—	—	—	−3.40 to 1.20
Indirect effect, PTQ (a3*b3)	−2.83	—	—	—	−5.80 to −0.57
Indirect effect, combined (a1*b1 + a2*b2 + a3*b3)	−6.07	—	—	—	−8.95 to −3.26
Total effect (a1*b1 + a2*b2 + a3*b3 + *c’*)	−9.59	—	—	—	−13.43 to −5.31

^a^BADS: Behavioral Activation for Depression Scale.

^b^Italicized text indicate *P*<.05.

^c^CDS: Cognitive Distortions Scale.

^d^PTQ: Perseverative Thinking Questionnaire.

^e^Not available.

## Discussion

### Principal Findings

The findings from this investigation contribute to the mHealth literature by providing convincing evidence that text-delivered CBT treatment can significantly and consistently reduce depressive symptoms in young adults. These findings also specify 3 treatment mechanisms that each explain a significant portion of the treatment effect when separately introduced into the mediation analysis models and even larger effects when combined. These results support the specification of 3 candidate therapeutic mechanisms of change within CBT-txt.

All 4 of our hypotheses were supported in this trial’s findings. Our primary hypothesis tested the efficacy of CBT-txt to reduce depressive symptoms, relative to the control condition. The results supported this hypothesis across all 3 months of the study, revealing a strong treatment effect. The mediation analyses also supported our hypotheses regarding the mechanisms of change within the CBT-txt treatment structure. Separate models showed that each mediator accounted for a substantial proportion of the CBT-txt treatment effect on depression. Models with all mediators included showed that, combined, these mechanisms explained 63% of the CBT-txt treatment effect, with changes in perseverative thinking showing the strongest independent indirect effect.

Although we did not experimentally test dosage effects in this trial, this study was a follow-up to our initial CBT-txt trial [9], where participants indicated wanting more texts and more detail on CBT. Thus, an important general research question was related to the treatment effect and acceptability of an 8-week versus a 4-week treatment. The direct treatment effect improved over the first trial (Cohen *d*=−0.63 to Cohen *d*=−0.76), and significant treatment effects were found at 3 months after enrollment compared with 1 month after enrollment in the initial 4-week trial. The largest changes in mechanism scores occurred during the first month of treatment. This may be an indication that these mechanisms are new or novel to participants and, as such, they may be more likely to engage with the treatment to quickly reduce their MDD symptoms. As noted, acceptability and satisfaction improved compared with the first trial. Specifically, the content was deemed helpful and the number of days of treatment and the number of texts per day appeared acceptable, supporting the increase in treatment dosage and thus aligning CBT-txt with the average length of mHealth treatment for depression.

Finally, these findings provide further support for recruiting young adults with depression via Facebook and Instagram, providing an efficient pathway toward engaging this hard-to-reach and underserved population. We recruited a sample of 103 participants in 7 weeks with excellent rates of retention, satisfaction, and engagement. These findings also support the use of CBT-txt as part of a continuum of care. For example, CBT-txt could serve as a form of pretreatment while individuals are placed on waitlists to be seen by clinicians. This approach could quickly reduce the severity of symptoms, particularly for individuals who are experiencing moderate to severe depressive symptoms. Targeting those with the most severe depression with CBT-txt may provide rapid symptom relief, which could then be followed up by a clinician. For some, CBT-txt may be enough; for others, it may serve as a jump start to their treatment and others may find it useful to combine CBT-txt while seeing a clinician for therapy. Supporting this latter idea, it is noteworthy that 278 participants were excluded from the study for reporting current or recent treatment for depression, suggesting that some young adults may recognize a need for supplemental options to traditional depression treatment. Social media recruitment may also reach young adults seeking preventive or early intervention services. Of note, 110 participants were not eligible for the study, as they reported only minimal (25/110, 22.7%) or mild (85/110, 77.3%) depression symptoms on the PHQ-9.

### Limitations

The study results should be considered in light of the following limitations. First, although this study was structured as a follow-up pilot study, having a larger sample size and longer follow-up periods may strengthen confidence in the findings. Second, the sample was 84% female, limiting the generalization across biological sexes. Although the rates of major depressive episodes are double for female young adults compared with male young adults (22.9% vs 11.1%) [1], the sample imbalance raises a methodological question as to how best to engage male young adults in treatment. More research is needed that tests varying recruitment methods, branding, and advertising content, including images and language, to engage more male young adults. Third, the control condition was a nonactive comparator and a waitlist control. The study findings could be strengthened by comparing CBT-txt against an active comparator. Fourth, experimental dosage testing could be conducted in future studies to determine the most clinically useful, acceptable, and efficient dosage. A larger multiarm study to test varying dosage levels and associated outcomes could be clinically and scientifically useful. Fifth, participants were not confirmed to have an MDD diagnoses, as we used the PHQ-9 as a screening and inclusion criterion. Finally, the sample was limited to individuals without SI. Given that this is very common among patients with MDD, this exclusion criterion altered the composition of the sample. Future studies with a more robust clinical infrastructure are needed to safely enroll patients with MDD and SI. Mobile interventions are not a replacement for standard depression treatment but may be used to quickly reduce symptoms while patients are on the waiting list, as an adjunct treatment component, or as part of a stepped-care model.

The results of this trial provide further support for CBT-txt as an efficacious, efficient, and reliable form of treatment to address depressive symptomatology among young adults. To the best of our knowledge, CBT-txt is unique in its SMS text message–delivered modality and in its strong clinical evidence supporting efficacy. While acknowledging the study limitations and the potential treatment effect fluctuations due to the sample size, these results build upon our first trial’s promising findings and provide more empirical evidence for the treatment format, delivery modality, and clinical possibilities of using CBT-txt at scale.

## Appendix

Multimedia Appendix 1 Full model results for latent change score mediations.

Multimedia Appendix 2 CONSORT eHEALTH checklist (V 1.6.2).

## Abbreviations

BDI-II: Beck Depression Inventory-II
CBT: cognitive behavioral therapy
CBT-txt: SMS text message–delivered cognitive behavioral therapy
CDS: Cognitive Distortions Scale
CONSORT: Consolidated Standards of Reporting Trials
LCS: latent change score
MDD: major depressive disorder
mHealth: mobile health
PHQ-9: Patient Health Questionnaire–9
PTQ: Perseverative Thinking Questionnaire
RCI: reliable change index
SI: suicidal ideation
