# Supplementary material for: Testing Mechanisms of Change for Text Message–Delivered Cognitive Behavioral Therapy: Randomized Clinical Trial for Young Adult Depression
Source: JMIR Mhealth Uhealth. 2023 Jul 11;11:e45186. doi: 10.2196/45186 (PMC10369163; doi:10.2196/45186)
Supplement: Multimedia Appendix 1 [file mhealth_v11i1e45186_app1.docx]

**Supplemental Materials for**

Comparing Dosage and Testing Mechanisms for Text-Message Delivered Cognitive Behavioral Therapy for Young Adult Depression: A Follow-up Randomized Clinical Trial

Michael J. Mason

J. Douglas Coatsworth

Nikola Zaharakis

Michael A. Russell

Aaron Brown

Sydney McKinstry

| ***Table S1****. Latent change score model results testing CBT-txt efficacy for behavioral activation* | | | | | | |
| --- | --- | --- | --- | --- | --- | --- |
|  | Est | SE | Z | p | Lower CL | Upper CL |
| *Baseline Activation Level* |  |  |  |  |  |  |
| Intercept | 0.00 | 0.66 | 0.00 | 1.000 | -1.29 | 1.29 |
| CBT-txt | -1.92 | 1.32 | -1.46 | 0.145 | -4.50 | 0.66 |
| *Change (Baseline - 1-month)* |  |  |  |  |  |  |
| Intercept | **5.20** | 0.71 | 7.35 | <0.001 | 3.82 | 6.59 |
| CBT-txt | **7.28** | 1.43 | 5.09 | <0.001 | 4.48 | 10.09 |
| Baseline Activation Level | **-0.57** | 0.11 | -5.40 | <0.001 | -0.77 | -0.36 |
| *Change (1-month - 2-month)* |  |  |  |  |  |  |
| Intercept | **3.04** | 0.80 | 3.80 | <0.001 | 1.47 | 4.62 |
| CBT-txt | *2.48* | 1.45 | 1.70 | 0.088 | -0.37 | 5.32 |
| 1-month Activation Level | **-0.39** | 0.09 | -4.27 | <0.001 | -0.57 | -0.21 |
| Baseline Activation Level | -0.05 | 0.10 | -0.51 | 0.608 | -0.25 | 0.15 |
| *Change (2-month - 3-month)* |  |  |  |  |  |  |
| Intercept | **2.08** | 0.82 | 2.54 | 0.011 | 0.48 | 3.68 |
| CBT-txt | *2.48* | 1.41 | 1.77 | 0.077 | -0.27 | 5.24 |
| 2-month Activation Level | **-0.34** | 0.08 | -4.10 | <0.001 | -0.50 | -0.18 |
| Baseline Activation Level | 0.13 | 0.10 | 1.38 | 0.168 | -0.06 | 0.32 |

| *Table S2. Latent change score model results testing CBT-txt efficacy for distortions* | | | | | | |
| --- | --- | --- | --- | --- | --- | --- |
|  | Est | SE | Z | p | Lower CL | Upper CL |
| *Baseline Distortions Level* |  |  |  |  |  |  |
| Intercept | 0.00 | 1.99 | 0.00 | 1.000 | -3.91 | 3.91 |
| CBT-txt | -5.60 | 3.99 | -1.40 | 0.160 | -13.41 | 2.22 |
| *Change (Baseline - 1-month)* |  |  |  |  |  |  |
| Intercept | **-9.25** | 1.75 | -5.28 | <0.001 | -12.69 | -5.82 |
| CBT-txt | **-10.29** | 3.53 | -2.92 | 0.004 | -17.20 | -3.38 |
| Baseline Distortions Level | **-0.33** | 0.09 | -3.67 | <0.001 | -0.50 | -0.15 |
| *Change (1-month - 2-month)* |  |  |  |  |  |  |
| Intercept | **-7.30** | 1.43 | -5.09 | <0.001 | -10.11 | -4.49 |
| CBT-txt | **-11.54** | 2.63 | -4.38 | <0.001 | -16.70 | -6.38 |
| 1-month Distortions Level | **-0.32** | 0.07 | -4.45 | <0.001 | -0.46 | -0.18 |
| Baseline Distortions Level | 0.11 | 0.08 | 1.39 | 0.165 | -0.05 | 0.27 |
| *Change (2-month - 3-month)* |  |  |  |  |  |  |
| Intercept | **-4.17** | 2.01 | -2.07 | 0.038 | -8.12 | -0.22 |
| CBT-txt | -3.68 | 3.61 | -1.02 | 0.308 | -10.75 | 3.39 |
| 2-month Distortions Level | **-0.31** | 0.09 | -3.35 | 0.001 | -0.49 | -0.13 |
| Baseline Distortions Level | 0.11 | 0.09 | 1.15 | 0.249 | -0.08 | 0.29 |

| *Table S3. Latent change score model results testing CBT-txt efficacy for perseverative thinking* | | | | | | |
| --- | --- | --- | --- | --- | --- | --- |
|  | Est | SE | Z | p | Lower CL | Upper CL |
| *Baseline Perseveration Level* |  |  |  |  |  |  |
| Intercept | 0.00 | 1.02 | 0.00 | 1.000 | -2.00 | 2.00 |
| CBT-txt | -2.75 | 2.04 | -1.35 | 0.177 | -6.74 | 1.24 |
| *Change (Baseline - 1-month)* |  |  |  |  |  |  |
| Intercept | **-5.43** | 0.99 | -5.48 | <0.001 | -7.37 | -3.49 |
| CBT-txt | **-7.16** | 2.00 | -3.59 | <0.001 | -11.08 | -3.25 |
| Baseline Perseveration Level | **-0.35** | 0.10 | -3.64 | <0.001 | -0.53 | -0.16 |
| *Change (1-month - 2-month)* |  |  |  |  |  |  |
| Intercept | **-1.74** | 0.79 | -2.20 | 0.028 | -3.29 | -0.19 |
| CBT-txt | **-4.85** | 1.48 | -3.27 | 0.001 | -7.75 | -1.94 |
| 1-month Perseveration Level | **-0.21** | 0.07 | -3.00 | 0.003 | -0.35 | -0.07 |
| Baseline Perseveration Level | 0.02 | 0.08 | 0.18 | 0.854 | -0.14 | 0.17 |
| *Change (2-month - 3-month)* |  |  |  |  |  |  |
| Intercept | **-2.99** | 0.86 | -3.46 | 0.001 | -4.68 | -1.29 |
| CBT-txt | -1.47 | 1.68 | -0.87 | 0.383 | -4.77 | 1.83 |
| 2-month Perseveration Level | **-0.17** | 0.07 | -2.29 | 0.022 | -0.31 | -0.02 |
| Baseline Perseveration Level | 0.09 | 0.08 | 1.15 | 0.252 | -0.07 | 0.25 |

| *Table S4. Latent change score mediation model results testing CBT-txt efficacy for depression through behavioral activation* | | | | | | |
| --- | --- | --- | --- | --- | --- | --- |
|  | Est | SE | Z | p | Lower CL | Upper CL |
| *Activation Change (Baseline - 3-month)* |  |  |  |  |  |  |
| Intercept | **6.17** | 0.82 | 7.53 | <0.001 | 4.68 | 7.89 |
| CBT-txt (*path* *a*) | **7.03** | 1.68 | 4.20 | <0.001 | 3.75 | 10.30 |
| Baseline Activation Level | **-0.73** | 0.14 | -5.30 | <0.001 | -1.03 | -0.48 |
| Baseline Depression Level | -0.01 | 0.09 | -0.07 | 0.948 | -0.18 | 0.18 |
| *Depression Change (Baseline - 3-month)* |  |  |  |  |  |  |
| Intercept | **-7.73** | 1.11 | -6.98 | <0.001 | -9.99 | -5.68 |
| Activation Change (Baseline - 3-month) (*path b*) | **-0.75** | 0.11 | -6.78 | <0.001 | -0.96 | -0.53 |
| CBT-txt (*path c'*) | **-4.01** | 1.83 | -2.19 | 0.028 | -7.69 | -0.49 |
| Baseline Activation Level | -0.27 | 0.16 | -1.63 | 0.104 | -0.59 | 0.05 |
| Baseline Depression Level | **-0.55** | 0.12 | -4.71 | <0.001 | -0.78 | -0.33 |
|  |  |  |  |  |  |  |
| *Mediation Estimates* | Est | Lower CL | Upper CL | Sig |  |  |
| Indirect Effect (*a*b*) | **-5.27** | -8.69 | -2.54 | * |  |  |
| Total Effect (*a*b* + *c'*) | **-9.28** | -13.19 | -5.09 | * |  |  |
| Percent Mediated ([Indirect / Total]*100%) | 56.8% |  |  |  |  |  |

| *Table S5. Latent change score mediation model results testing CBT-txt efficacy for depression through cognitive distortions* | | | | | | |
| --- | --- | --- | --- | --- | --- | --- |
|  | Est | SE | Z | p | Lower CL | Upper CL |
| *Distortions Change (Baseline - 3-month)* |  |  |  |  |  |  |
| Intercept | **-13.54** | 1.99 | -6.79 | <0.001 | -17.48 | -9.70 |
| CBT-txt (*path* *a*) | **-15.98** | 4.06 | -3.94 | <0.001 | -23.99 | -7.92 |
| Baseline Distortions Level | **-0.40** | 0.13 | -3.21 | 0.001 | -0.66 | -0.17 |
| Baseline Depression Level | -0.39 | 0.26 | -1.49 | 0.137 | -0.92 | 0.11 |
| *Depression Change (Baseline - 3-month)* |  |  |  |  |  |  |
| Intercept | **-8.93** | 1.04 | -8.61 | <0.001 | -10.96 | -6.88 |
| Distortions Change (Baseline - 3-month) (*path b*) | **0.25** | 0.04 | 6.30 | <0.001 | 0.17 | 0.32 |
| CBT-txt (*path c'*) | **-5.67** | 2.11 | -2.69 | 0.007 | -9.85 | -1.58 |
| Baseline Distortions Level | **0.12** | 0.06 | 2.17 | 0.030 | 0.01 | 0.24 |
| Baseline Depression Level | **-0.58** | 0.14 | -4.29 | <0.001 | -0.87 | -0.34 |
|  |  |  |  |  |  |  |
| *Mediation Estimates* | Est | Lower CL | Upper CL | Sig |  |  |
| Indirect Effect (*a*b*) | **-4.01** | -6.51 | -2.04 | * |  |  |
| Total Effect (*a*b* + *c'*) | **-9.68** | -13.67 | -5.35 | * |  |  |
| Percent Mediated ([Indirect / Total]*100%) | 41.4 |  |  |  |  |  |

| *S6. Latent change score mediation model results testing CBT-txt efficacy for depression through perseverative thinking* | | | | | | |
| --- | --- | --- | --- | --- | --- | --- |
|  | Est | SE | Z | p | Lower CL | Upper CL |
| *Perseveration Change (Baseline - 3-month)* |  |  |  |  |  |  |
| Intercept | **-8.02** | 1.16 | -6.94 | <0.001 | -10.37 | -5.84 |
| CBT-txt (*path* *a*) | **-10.33** | 2.40 | -4.30 | <0.001 | -14.95 | -5.63 |
| Baseline Perseveration Level | **-0.50** | 0.16 | -3.16 | 0.002 | -0.83 | -0.21 |
| Baseline Depression Level | 0.09 | 0.17 | 0.50 | 0.616 | -0.26 | 0.41 |
| *Depression Change (Baseline - 3-month)* |  |  |  |  |  |  |
| Intercept | **-8.33** | 0.97 | -8.60 | <0.001 | -10.26 | -6.50 |
| Perseveration Change (Baseline - 3-month) (*path b*) | **0.50** | 0.06 | 8.43 | <0.001 | 0.38 | 0.61 |
| CBT-txt (*path c'*) | **-5.18** | 1.79 | -2.89 | 0.004 | -8.64 | -1.57 |
| Baseline Perseveration Level | 0.04 | 0.09 | 0.47 | 0.639 | -0.13 | 0.22 |
| Baseline Depression Level | **-0.57** | 0.12 | -4.93 | <0.001 | -0.80 | -0.35 |
|  |  |  |  |  |  |  |
| *Mediation Estimates* | Est | Lower CL | Upper CL | Sig |  |  |
| Indirect Effect (*a*b*) | **-5.12** | -8.05 | -2.66 | * |  |  |
| Total Effect (*a*b* + *c'*) | **-10.30** | -14.02 | -6.19 | * |  |  |
| Percent Mediated ([Indirect / Total]*100%) | 49.7 |  |  |  |  |  |
