# Supplementary material for: Testing Mechanisms of Change for Text Message–Delivered Cognitive Behavioral Therapy: Randomized Clinical Trial for Young Adult Depression
Source: JMIR Mhealth Uhealth. 2023 Jul 11;11:e45186. doi: 10.2196/45186 (PMC10369163; doi:10.2196/45186)
Supplement: Multimedia Appendix 2 [file mhealth_v11i1e45186_app2.pdf]

CONSORT-EHEALTH Checklist V1.6.2 Report	Manuscript Number	45186
**(based on CONSORT-EHEALTH V1.6), available at [http://tinyurl.com/consort-ehealth-v1-6].**		
**Date completed**		
4/12/2023 15:13:07		
**by**		
Michael J. Mason		
Testing Mechanisms of Change for Text-Message Delivered Cognitive Behavioral Therapy: A Randomized Clinical Trial for Young Adult Depression		
TITLE		
**1a-i) Identify the mode of delivery in the title**		
"Testing Mechanisms of Change for Text-Message Delivered Cognitive Behavioral Therapy: A Randomized Clinical Trial for Young Adult Depression"		
**1a-ii) Non-web-based components or important co-interventions in title**		
**1a-iii) Primary condition or target group in the title**		
"Testing Mechanisms of Change for Text-Message Delivered Cognitive Behavioral Therapy: A Randomized Clinical Trial for Young Adult Depression"		
ABSTRACT		
**1b-i) Key features/functionalities/components of the intervention and comparator in the METHODS section of the ABSTRACT**		
"Participants were randomized to CBT-txt or a waitlist control condition."		
**1b-ii) Level of human involvement in the METHODS section of the ABSTRACT**		
"Those assigned to the CBT-txt intervention condition received 474 fully automated texts, delivered every-other-day over a 64-day period, averaging 14.8 texts per treatment day."		
**1b-iii) Open vs. closed, web-based (self-assessment) vs. face-to-face assessments in the METHODS section of the ABSTRACT**		
"Assessments were web-based and occurred at baseline prior to randomization and at 1, 2, and 3 months post-enrollment."		
**1b-iv) RESULTS section in abstract must contain use data**		
**1b-v) CONCLUSIONS/DISCUSSION in abstract for negative trials**		
INTRODUCTION		
**2a-i) Problem and the type of system/solution**		
" Paradoxically, more young adults with depression report greater perceived unmet need for mental health services compared to other age groups.1 From 2011 to 2019, the most common reason young adults report not receiving treatment is cost, followed by 'not knowing where to go to receive treatment'.3 This unmet treatment need has been consistent over the last decade and places this group at increased risk for substance use disorders, possibly indicating self-medication with substances to reduce depressive symptoms.4 Creative mental health treatment strategies are needed to reach young adults in order to address this unmet need. Digitally delivered mobile health treatments (or mHealth interventions), show promise for reaching young adults and serving as a clinical tool to address young adult depression. "		
**2a-ii) Scientific background, rationale: What is known about the (type of) system**		
"During 2020 young adults (ages 18 -25) in the United States experienced more Major Depressive Episodes than other age groups and were the least likely group to receive treatment for depression (e.g., talking with a health provider or taking prescription medication).[1] Even when rates for treatment of depression rise nationwide, rates for young adults' treatment of depression rise at a significantly lower rate.2"		
**Does your paper address CONSORT subitem 2b?**		
"Four hypotheses were tested for this follow-up study. The first hypothesis was that participants allocated to the treatment condition would show greater reductions in their depressive symptoms immediately following treatment (2 months post enrollment) and at the 3-month follow-up compared to participants in the waitlist control condition. Because treatment response has been linked to baseline symptom severity,9,14 we also tested whether treatment effects were moderated by baseline severity level. The second hypothesis was that treatment effects would be mediated by behavioral activation, such that increases in behavioral activation would reduce depressive symptoms in the treatment condition relative to controls. The third hypothesis was that treatment effects would be mediated by cognitive distortions, such that decreases in cognitive distortions would reduce depressive symptoms in the treatment condition relative to controls. The fourth hypothesis was that treatment effects would be mediated by perseverative thinking, such that decreases in perseverative thinking would reduce depressive symptoms in the treatment condition relative to controls. In addition to testing these four hypotheses, we were interested in comparing the results (effect sizes and treatment response over-time) from this 8-week intervention with the results from our prior trial of the 4-week intervention."		
METHODS		
**3a) CONSORT: Description of trial design (such as parallel, factorial) including allocation ratio**		
"The study design was a two-arm randomized clinical trial with participants allocated to either the experimental condition or the waitlist control condition. Randomization was automated by Qualtrics as part of the baseline survey. Randomization was stratified by gender using block randomization with a fixed block size of 10 to reduce bias during randomization and to ensure equal representation of gender across both conditions. "		
**3b) CONSORT: Important changes to methods after trial commencement (such as eligibility criteria), with reasons**		
no changes were made in this category		
**3b-i) Bug fixes, Downtimes, Content Changes**		
no changes were made in this category		
**4a) CONSORT: Eligibility criteria for participants**		
"Eligibility requirements were 1) age between 18 and 25; 2) score of at least 10 on the Patient Health Questionnaire-9,15 indicating at least moderate depressive symptom severity; 3) access to a smart phone; 4) fluent in English; 5) have not received treatment for depression the past three months; and 6), did not endorse suicidal ideation on the PHQ-9 measure. "		
**4a-i) Computer / Internet literacy**		
**4a-ii) Open vs. closed, web-based vs. face-to-face assessments:**		
"Interested individuals were directed to a study website where they would read more about the study and answer eligibility screening questions on their phones. Eligible individuals were instructed to complete the baseline survey on their phones where upon completion they were randomized to either the treatment or waitlist control condition. "		
**4a-iii) Information giving during recruitment**		
**4b) CONSORT: Settings and locations where the data were collected**		
"Participants were recruited using Facebook and Instagram age-targeted advertising for young adults residing within the U.S. Recruitment occurred over a 7-week period from July 6, 2022 through August 28, 2022. The study was advertised to all those within the study age range (18-25 years) with the aim of recruiting a geographic, racial/ethnic, and socioeconomic diverse sample by allowing all U.S. young adults using Facebook or Instagram to potentially see our advertisements. Interested individuals were directed to a study website where they would read more about the study and answer eligibility screening questions on their phones. "		
**4b-i) Report if outcomes were (self-)assessed through online questionnaires**		
"Interested individuals were directed to a study website where they would read more about the study and answer eligibility screening questions on their phones. Eligible individuals were instructed to complete the baseline survey on their phones where upon completion they were randomized to either the treatment or waitlist control condition. "		
**4b-ii) Report how institutional affiliations are displayed**		
**5) CONSORT: Describe the interventions for each group with sufficient details to allow replication, including how and when they were actually administered**		
**5-i) Mention names, credential, affiliations of the developers, sponsors, and owners**		
**5-ii) Describe the history/development process**		
**5-iii) Revisions and updating**		
**5-iv) Quality assurance methods**		

5-v) Ensure replicability by publishing the source code, and/or providing screenshots/screen-capture video, and/or providing flowcharts of the algorithms used

5-vi) Digital preservation

5-vii) Access

"One hundred and three young adults (ages 18 to 25) were recruited and enrolled into the three-month randomized clinical trial. Participants were recruited using Facebook and Instagram age-targeted advertising for young adults residing within the U.S. Recruitment occurred over a 7-week period from July 6, 2022 through August 28, 2022. The study was advertised to all those within the study age range (18-25 years) with the aim of recruiting a geographic, racial/ethnic, and socioeconomic diverse sample by allowing all U.S. young adults using Facebook or Instagram to potentially see our advertisements. Interested individuals were directed to a study website where they would read more about the study and answer eligibility screening questions on their phones. "

5-viii) Mode of delivery, features/functionalities/components of the intervention and comparator, and the theoretical framework

"CBT-txt was adapted from an evidenced-based, in-person, CBT treatment manual17 shown to be effective in reducing depressive symptoms18–20 and adaptable to digital formats.21 CBT-txt focuses on empowering participants to understand how thoughts, activities, and other people affect their moods. CBT-txt is a fully automated text-message delivered (SMS) program that initiates a conversation at pre-determined times and requires participant response to activate the subsequent text message. Tailored messages are sent based upon participant responses or ratings of their depression. For example, a participant may be asked about their engagement with the CBT skills and are given 3 response choices, a, b, or c. Each response option activates a different CBT-txt message providing tailored responses. The treatment provides individualized, text conversations every other day over the course of the intervention.

Theoretical Underpinnings of CBT-txt. The theory underlying CBT-txt is the Generic Cognitive Model which specifies common cognitive and behavioral processes associated with disorders such as depression.22 These processes manifest along a continuum from normal adaptive functioning to psychopathology. As individuals experience environmental, psychological, and social stimuli, their attentional response system is activated to determine an adaptive response. The attentional response activates schemas, defined as internally stored representations of stimuli which form underlying structures for organizing perceptions of the world.23 When the schema is maladaptive or is disproportionate relative to the stimuli, the individual experiences psychological problems that can escalate into a psychiatric disorder.

Clinical Structure of CBT-txt. We expanded our initial 4-week text-delivered CBT-txt treatment9 into an 8-week intervention. CBT-txt intervention content is guided by CBT core mechanisms11 and is sequenced using the Muñoz et al in-person treatment manual. CBT-txt is organized around eight topical areas delivered across 8 weeks as indicated in Table 1: 1) introduction to CBT, 2) automatic thoughts, 3) behavioral activation, 4) automatic thoughts and health, 5) perseverative thinking, 6) cognitive distortions, 7) more on behavioral activation, and 8), social support and summary."

5-ix) Describe use parameters

5-x) Clarify the level of human involvement

5-xi) Report any prompts/reminders used

Not relevant to this study

5-xii) Describe any co-interventions (incl. training/support)

Not relevant to this study

6a) CONSORT: Completely defined pre-specified primary and secondary outcome measures, including how and when they were assessed

"Measures

Demographics. Participant age, sex, race/ethnicity, socioeconomic status, family history of depression, and current use of antidepressant medication was collected at baseline assessment.

Acceptability and Engagement. Participants' acceptability of the intervention was measured via participant-reported satisfaction with the intervention, as well as passively collected engagement with the intervention content and features. Satisfaction was assessed using a measure developed in our past work.28 Two items assessed whether the number of days on which texts were received and the number of texts received each day were (1) too few, (2) just right, or (3), too many. An additional two items measured whether the texts were easy to understand and complete, and whether the 4MOOD booster messages were a helpful option. In addition, five items measured perceptions of the helpfulness of intervention content (Cronbach alpha = 0.88), and six items assessed participants' practice of the intervention skills taught (Cronbach alpha = 0.78). All of these items were rated on a 5-point Likert scale (1=Strongly Disagree, 5=Strongly Agree). Engagement with the intervention was measured using data passively collected that was programmed to be automatically gathered during the administration of the intervention, including the number of intervention texts a participant responded to each day and the number of booster text messages requested.

Screen of Depression Symptoms. The Patient Health Questionnaire-9 was used to determine eligibility.15 The PHQ-9 is a questionnaire consisting of all nine criteria for major depressive disorder. Responses range from "Not at all" (0) to "Nearly every day" (3). Item scores are summed for a total score ranging from 0 to 27. The PHQ-9 has good validity, test–retest reliability, and internal consistency. Cronbach's alpha in the present sample was 0.50.

Depression Symptoms and Severity. Baseline and follow-up assessment of depression severity was assessed with the Beck Depression Inventory II.29 There are 21-items, each corresponding with a symptom of depression, and possible scores on each item range from zero to three. Scores are summed to give a single severity score. A score of 0-13 is considered minimal depressive symptoms, 14-19 indicates mild depressive symptoms, 20-28 indicates moderate depressive symptoms, and scores of 29 and above indicate severe depressive symptoms. Cronbach's alpha in the present sample at each assessment was 0.83, 0.89, 0.92, 0.92.

Behavioral Activation. Behavioral activation was measured using the Behavioral Activation for Depression Scale, Short Form.30 The BADS-SF is a nine-item scale used to assess activation towards goals during treatment for depression. Items are scored from zero to six, and higher scores indicate more activation towards goals and less avoidance of tasks. Cronbach's alpha in the present sample at each assessment was 0.70, 0.70, 0.82, 0.81.

Perseverative Thinking. Repetitive negative thinking was measured using the Perseverative Thinking Questionnaire.31 The PTQ is a fifteen-item questionnaire used to characterize respondent thinking about negative experiences or problems. Items are scored on a 5-point scale from 0 = Never to 4 = Almost always, and are summed with higher scores indicating more repetitive negative thinking. Cronbach's alpha in the present sample at each assessment was 0.94, 0.95, 0.93, 0.97.

Cognitive Distortion. Cognitive distortion was measured using the Cognitive Distortion Scale.32 The CDS assesses 10 types of thinking biases (e.g., catastrophizing, all-or-nothing thinking). Participants rate the frequency of their use of each type of thinking. Items are scored on a 7-point scale from 0 = Never to 7 = All the time, and are summed with higher scores indicating more cognitive distortion. Cronbach's alpha in the present sample at each assessment was 0.92, 0.94, 0.93, 0.95."

6a-i) Online questionnaires: describe if they were validated for online use and apply CHERRIES items to describe how the questionnaires were designed/deployed

6a-ii) Describe whether and how "use" (including intensity of use/dosage) was defined/measured/monitored

6a-iii) Describe whether, how, and when qualitative feedback from participants was obtained

6b) CONSORT: Any changes to trial outcomes after the trial commenced, with reasons

"Participants were recruited using Facebook and Instagram age-targeted advertising for young adults residing within the U.S. Recruitment occurred over a 7-week period from July 6, 2022 through August 28, 2022. The study was advertised to all those within the study age range (18-25 years) with the aim of recruiting a geographic, racial/ethnic, and socioeconomic diverse sample by allowing all U.S. young adults using Facebook or Instagram to potentially see our advertisements. Interested individuals were directed to a study website where they would read more about the study and answer eligibility screening questions on their phones. "

7a) CONSORT: How sample size was determined

7a-i) Describe whether and how expected attrition was taken into account when calculating the sample size

7b) CONSORT: When applicable, explanation of any interim analyses and stopping guidelines

"Measures
Demographics. Participant age, sex, race/ethnicity, socioeconomic status, family history of depression, and current use of antidepressant medication was collected at baseline assessment.
Acceptability and Engagement. Participants' acceptability of the intervention was measured via participant-reported satisfaction with the intervention, as well as passively collected engagement with the intervention content and features. Satisfaction was assessed using a measure developed in our past work.28 Two items assessed whether the number of days on which texts were received and the number of texts received each day were (1) too few, (2) just right, or (3), too many. An additional two items measured whether the texts were easy to understand and complete, and whether the 4MOOD booster messages were a helpful option. In addition, five items measured perceptions of the helpfulness of intervention content (Cronbach alpha = 0.88), and six items assessed participants' practice of the intervention skills taught (Cronbach alpha = 0.78). All of these items were rated on a 5-point Likert scale (1=Strongly Disagree, 5=Strongly Agree). Engagement with the intervention was measured using data passively collected that was programmed to be automatically gathered during the administration of the intervention, including the number of intervention texts a participant responded to each day and the number of booster text messages requested.
Screen of Depression Symptoms. The Patient Health Questionnaire-9 was used to determine eligibility.15 The PHQ-9 is a questionnaire consisting of all nine criteria for major depressive disorder. Responses range from "Not at all" (0) to "Nearly every day" (3). Item scores are summed for a total score ranging from 0 to 27. The PHQ-9 has good validity, test–retest reliability, and internal consistency. Cronbach's alpha in the present sample was 0.50.
Depression Symptoms and Severity. Baseline and follow-up assessment of depression severity was assessed with the Beck Depression Inventory II.29 There are 21-items, each corresponding with a symptom of depression, and possible scores on each item range from zero to three. Scores are summed to give a single severity score. A score of 0-13 is considered minimal depressive symptoms, 14-19 indicates mild depressive symptoms, 20-28 indicates moderate depressive symptoms, and scores of 29 and above indicate severe depressive symptoms. Cronbach's alpha in the present sample at each assessment was 0.83, 0.89, 0.92, 0.92.
Behavioral Activation. Behavioral activation was measured using the Behavioral Activation for Depression Scale, Short Form.30 The BADS-SF is a nine-item scale used to assess activation towards goals during treatment for depression. Items are scored from zero to six, and higher scores indicate more activation towards goals and less avoidance of tasks. Cronbach's alpha in the present sample at each assessment was 0.70, 0.70, 0.82, 0.81.
Perseverative Thinking. Repetitive negative thinking was measured using the Perseverative Thinking Questionnaire.31 The PTQ is a fifteen-item questionnaire used to characterize respondent thinking about negative experiences or problems. Items are scored on a 5-point scale from 0 = Never to 4 = Almost always, and are summed with higher scores indicating more repetitive negative thinking. Cronbach's alpha in the present sample at each assessment was 0.94, 0.95, 0.93, 0.97.
Cognitive Distortion. Cognitive distortion was measured using the Cognitive Distortion Scale.32 The CDS assesses 10 types of thinking biases (e.g., catastrophizing, all-or-nothing thinking). Participants rate the frequency of their use of each type of thinking. Items are scored on a 7-point scale from 0 = Never to 7 = All the time, and are summed with higher scores indicating more cognitive distortion. Cronbach's alpha in the present sample at each assessment was 0.92, 0.94, 0.93, 0.95."

8a) CONSORT: Method used to generate the random allocation sequence

"Eligible individuals were instructed to complete the baseline survey on their phones where upon completion they were randomized to either the treatment or waitlist control condition. Randomization was automated by Qualtrics as part of the baseline survey. Randomization was stratified by gender using block randomization with a fixed block size of 10 to reduce bias during randomization and to ensure equal representation of gender across both conditions."

8b) CONSORT: Type of randomisation; details of any restriction (such as blocking and block size)

"Randomization was automated by Qualtrics as part of the baseline survey. Randomization was stratified by gender using block randomization with a fixed block size of 10 to reduce bias during randomization and to ensure equal representation of gender across both conditions. "

9) CONSORT: Mechanism used to implement the random allocation sequence (such as sequentially numbered containers), describing any steps taken to conceal the sequence until interventions were assigned

"Randomization was automated by Qualtrics as part of the baseline survey. Randomization was stratified by gender using block randomization with a fixed block size of 10 to reduce bias during randomization and to ensure equal representation of gender across both conditions. "

10) CONSORT: Who generated the random allocation sequence, who enrolled participants, and who assigned participants to interventions

"Randomization was automated by Qualtrics as part of the baseline survey. Randomization was stratified by gender using block randomization with a fixed block size of 10 to reduce bias during randomization and to ensure equal representation of gender across both conditions. "

11a) CONSORT: Blinding - If done, who was blinded after assignment to interventions (for example, participants, care providers, those assessing outcomes) and how

11a-i) Specify who was blinded, and who wasn't

No blinding was used as participants received text-delivered treatment or assessment only.

11a-ii) Discuss e.g., whether participants knew which intervention was the "intervention of interest" and which one was the "comparator"

11b) CONSORT: If relevant, description of the similarity of interventions

Not relevant for this trial

12a) CONSORT: Statistical methods used to compare groups for primary and secondary outcomes

"Statistical analyses
Latent change score (LCS) modeling. LCS analyses was conducted in a structural equation modeling framework using the lavaan package in R. LCS was used instead of more commonly used modeling methods like latent growth modeling and repeated measures ANOVA because it offers two unique capabilities. First, it allows estimation of wave-to-wave change in mediators and outcomes adjusted for previous levels, which controls for regression to the mean. Second, it does not force the pattern of change to follow a pre-specified shape; wave-to-wave changes are allowed to freely vary across time.33,34 Latent change scores are created by (a) specifying an autoregression of each score on its immediately previous time point while fixing the coefficient to 1 and (b) specifying a latent factor with a loading of 1 on the current time point to capture the difference. These latent change scores are then used as dependent variables in regressions testing for CBT-txt intervention effects. BDI-II depression scores were centered on their pretreatment mean; CBT-txt was also centered (CBT-txt = .51, Control = -.49). This centering strategy facilitates interpretation of model intercepts as change from the previous month for a person with average pretreatment levels of depression, averaged across conditions. All latent change score regressions controlled for both pretreatment levels of depression and prior month levels of depression to account for the likely association between pretreatment severity and post-treatment change. Mediation was also tested in LCS models. Latent change scores were created for (a) baseline-to-3-month change in the mediator, (BADS, CDS, PTQ in separate models) and (b) baseline-to-3-month change in the outcome (BDI-II). Pretreatment mediator and BDI-II scores at baseline were included as covariates in the model. Change in mediators was regressed on CBT-txt (and baseline covariates) to create the a path; change in BDI-II was regressed on change in the mediator (the b path) and CBT-txt (the direct effect or c' path). The significance of the indirect effect (a*b) was tested using bias corrected bootstrapped confidence intervals with 10,000 bootstrap draws. The indirect effect tests the hypothesis that CBT-txt influences BDI-II through the mediator.
"

12a-i) Imputation techniques to deal with attrition / missing values

Our missingness was 3 participants, so not adjustments were necessary

12b) CONSORT: Methods for additional analyses, such as subgroup analyses and adjusted analyses

"Statistical analyses
Latent change score (LCS) modeling. LCS analyses was conducted in a structural equation modeling framework using the lavaan package in R. LCS was used instead of more commonly used modeling methods like latent growth modeling and repeated measures ANOVA because it offers two unique capabilities. First, it allows estimation of wave-to-wave change in mediators and outcomes adjusted for previous levels, which controls for regression to the mean. Second, it does not force the pattern of change to follow a pre-specified shape; wave-to-wave changes are allowed to freely vary across time.33,34 Latent change scores are created by (a) specifying an autoregression of each score on its immediately previous time point while fixing the coefficient to 1 and (b) specifying a latent factor with a loading of 1 on the current time point to capture the difference. These latent change scores are then used as dependent variables in regressions testing for CBT-txt intervention effects. BDI-II depression scores were centered on their pretreatment mean; CBT-txt was also centered (CBT-txt = .51, Control = -.49). This centering strategy facilitates interpretation of model intercepts as change from the previous month for a person with average pretreatment levels of depression, averaged across conditions. All latent change score regressions controlled for both pretreatment levels of depression and prior month levels of depression to account for the likely association between pretreatment severity and post-treatment change. Mediation was also tested in LCS models. Latent change scores were created for (a) baseline-to-3-month change in the mediator, (BADS, CDS, PTQ in separate models) and (b) baseline-to-3-month change in the outcome (BDI-II). Pretreatment mediator and BDI-II scores at baseline were included as covariates in the model. Change in mediators was regressed on CBT-txt (and baseline covariates) to create the a path; change in BDI-II was regressed on change in the mediator (the b path) and CBT-txt (the direct effect or c' path). The significance of the indirect effect (a*b) was tested using bias corrected bootstrapped confidence intervals with 10,000 bootstrap draws. The indirect effect tests the hypothesis that CBT-txt influences BDI-II through the mediator.
"

RESULTS

13a) CONSORT: For each group, the numbers of participants who were randomly assigned, received intended treatment, and were analysed for the primary outcome

Our analytic sample was 100, 53 in control and 47 in treatment condition

13b) CONSORT: For each group, losses and exclusions after randomisation, together with reasons

3 participants were lost to follow-up

13b-i) Attrition diagram

14a) CONSORT: Dates defining the periods of recruitment and follow-up

"Participants were recruited using Facebook and Instagram age-targeted advertising for young adults residing within the U.S. Recruitment occurred over a 7-week period from July 6, 2022 through August 28, 2022. The study was advertised to all those within the study age range (18-25 years) with the aim of recruiting a geographic, racial/ethnic, and socioeconomic diverse sample by allowing all U.S. young adults using Facebook or Instagram to potentially see our advertisements. "		
**14a-i) Indicate if critical "secular events" fell into the study period**		
**14b) CONSORT: Why the trial ended or was stopped (early)**		
The trial did not end early		
**15) CONSORT: A table showing baseline demographic and clinical characteristics for each group**		
"Our sample size was 103. Participants' mean age was 22 (SD = 2.2), 84% were female, and they resided in 34 different states in the U.S and the District of Columbia. The sample was 63.7% White, 15.7% Asian, 8.8% more than one race, 7.8% Hispanic/Latino, and 3.9% Black/African American. While not used analytically, college student status and childhood socioeconomic status (Has your family ever received food assistance, such as free or reduced lunch, or SNAP benefits?), and past and current use of anti-depression medication is described in order to characterize the sample. Slightly more than half (54.5%) were not enrolled in college part- or full-time. Approximately one-third (34%) endorsed a family history of food assistance or SNAP benefits. Forty-three percent of participants had been prescribed antidepressant medication at some time in their life, but only 20% were currently prescribed antidepressant medication. Figure 1 provides details on the enrollment and allocation process via study CONSORT diagram. "		
**15-i) Report demographics associated with digital divide issues**		
We have no data about digital divide. Our sample was diverse except for sex which we discuss in the limitation section		
**16a) CONSORT: For each group, number of participants (denominator) included in each analysis and whether the analysis was by original assigned groups**		
**16-i) Report multiple "denominators" and provide definitions**		
We don't have these data at this point. It may be that further analysis could produce a secondary paper to cover this issue		
**16-ii) Primary analysis should be intent-to-treat**		
**17a) CONSORT: For each primary and secondary outcome, results for each group, and the estimated effect size and its precision (such as 95% confidence interval)**		
"CBT-txt main effect on depression. Figure 2 shows the BDI means over time for CBT-txt and control groups. Significant treatment-control group differences were seen at each of the three post-enrollment follow-ups (P < .001 at each follow-up). No treatment-control group difference was seen in pretreatment depression, supporting balance across groups in baseline levels. Table 3 shows the results of the LCS model testing whether monthly changes differed between CBT-txt and control groups. At each of the three follow-ups, young adults in the CBT-txt group showed significantly larger decreases in depression than those in the control group, producing a medium/large effect size (Cohen's d =.76). LCS mediation results. Table 4 shows a, b, c', indirect and total effects for mediation models testing indirect effects of CBT-txt on change in depression through change in the three hypothesized mechanisms (Supplemental Tables 4-6 show full model results for latent change score mediations). Significant direct and indirect effects of CBT-txt were seen on depression in all three models. CBT-txt appeared to lead to greater increases in behavioral activation and greater decreases in cognitive distortions and perseverative thinking across the three-month follow-up period, which were then associated with larger baseline-to-three-month decreases in depression. The size of indirect effects was substantial: 57%, 41%, and 50% of the CBT-txt effects on change in depression were mediated by change in behavioral activation, cognitive distortions, and perseverative thinking, respectively. "		
**17a-i) Presentation of process outcomes such as metrics of use and intensity of use**		
**17b) CONSORT: For binary outcomes, presentation of both absolute and relative effect sizes is recommended**		
Our results were not binary outcomes		
**18) CONSORT: Results of any other analyses performed, including subgroup analyses and adjusted analyses, distinguishing pre-specified from exploratory**		
"Clinical Significance. We used two measures of clinical significance. First, the number of participants at the end of the trial with high-end-state functioning as defined by Jacobson and Truax (1991) was measured. High-end-state functioning is defined as a participant having a BDI-II score between 0 and 13. By examining the BDI-II severity levels based on total scores (none to minimal depressive symptoms = 0-13; mild depressive symptoms = 14-19; moderate depressive symptoms = 20-28; and severe depressive symptoms = 29-63) treatment responses can be understood clinically. Thus, scores between 0 and 13 represent high-end-state functioning due to the elimination of symptoms used to classify participants with MDD. Over half of the treatment group, 53% (n=25) moved to the "none to minimal" category, compared to 15% (n=8) of the control group. Second, a Reliable Change Index (RCI) which is a measure of how much change has occurred during the course of treatment (Jacobson & Trux, 1991). Our study produced an RCI of 4.46 with 95% confidence, twice the minimal required RCI of 1.96. This RCI represents 2 standard deviations of clinical change for the treatment group based on baseline to the 3-month follow-up BDI-II scores. Figure 4 illustrates the depression severity level percentages at the 3-month assessment for comparison by experimental conditions. "		
**18-i) Subgroup analysis of comparing only users**		
**19) CONSORT: All important harms or unintended effects in each group**		
No harms or unintended effects were found as a result of this trial		
**19-i) Include privacy breaches, technical problems**		
**19-ii) Include qualitative feedback from participants or observations from staff/researchers**		
DISCUSSION		
**20) CONSORT: Trial limitations, addressing sources of potential bias, imprecision, multiplicity of analyses**		
**20-i) Typical limitations in ehealth trials**		
"Mobile interventions are not a replacement for standard depression treatment, but may be used to quickly reduce symptoms while patients are on waiting list, as an adjunct treatment component, or as part of a stepped-care model. "		
**21) CONSORT: Generalisability (external validity, applicability) of the trial findings**		
**21-i) Generalizability to other populations**		
**21-ii) Discuss if there were elements in the RCT that would be different in a routine application setting**		
**22) CONSORT: Interpretation consistent with results, balancing benefits and harms, and considering other relevant evidence**		
**22-i) Restate study questions and summarize the answers suggested by the data, starting with primary outcomes and process outcomes (use)**		
"Discussion<br>The findings from this investigation contribute to the mHealth literature by providing convincing evidence that text-delivered CBT treatment can significantly and consistently, reduce depressive symptoms in young adults. These findings also specify three treatment mechanisms that each explain a significant portion of the treatment effect when separately introduced into the mediation analysis models. These results support the specification of three candidate therapeutic mechanisms of change within CBT-txt."		
**22-ii) Highlight unanswered new questions, suggest future research**		
Other information		
**23) CONSORT: Registration number and name of trial registry**		
"The study was registered in clinical trials.gov under the identifier NCT05055284. "		
**24) CONSORT: Where the full trial protocol can be accessed, if available**		
"The study was registered in clinical trials.gov under the identifier NCT05055284. "		
**25) CONSORT: Sources of funding and other support (such as supply of drugs), role of funders**		
"This research was funded by the Betsey R. Bush endowment for<br>Children and Families at Risk, and the endowment for Behavioral<br>Health Research, Center for Behavioral Health Research, College<br>of Social Work, University of Tennessee."		
**X26-i) Comment on ethics committee approval**		
**x26-ii) Outline informed consent procedures**		
**X26-iii) Safety and security procedures**		
**X27-i) State the relation of the study team towards the system being evaluated**		
